# Expression of interleukin 6 (IL-6) correlates with oestrogen receptor in human breast carcinoma

**DOI:** 10.1038/sj.bjc.6690394

**Published:** 1999-05-01

**Authors:** G Fontanini, D Campani, M Roncella, D Cecchetti, S Calvo, A Toniolo, F Basolo

**Affiliations:** Department of Oncology, Divisions of Pathologyand Surgery, University of Pisa, 57 Via Roma, 56126, Pisa, Italy; Department of Clinical and Biological Sciences, University of Pavia, Varese, Italy

**Keywords:** cytokines, IL-6, breast cancer, ER, PgR, p53, Ki67

## Abstract

Multifunctional cytokines play important and only partially defined roles in mammary tumour development and progression. Normal human mammary epithelial cells constitutively produce interleukin 6 (IL-6), IL-8 and a non-secreted form of tumour necrosis factor. Transformation of mammary epithelial cells by different oncogenes is frequently associated with alterations of cytokine/growth factor production and responsiveness. In the present study we analysed the expression of IL-6 in 149 cases of invasive breast carcinoma and the data have been correlated with clinico-pathological variables including tumour size, histological grade, nodal status, and oestrogen and progesterone receptors, Ki67 and p53, protein expression. Though the majority of breast carcinomas expressed at least low levels of immunoreactive IL-6, we found that expression of this cytokine was inversely associated with histological tumour grade (*P* = 0.0017), but not with tumour size and nodal status. Ki67 positivity was inversely correlated with IL-6 expression (*P* = 0.027). Among biological parameters analysed, a direct association was found between the percentage of IL-6-positive cells and that of oestrogen (*P* = 0.00005) and progesterone (*P* = 0.025) receptor-positive cells. No correlation was observed between IL-6 and p53 protein expression. These data indicate that down-regulation of IL-6 is associated with highly malignant mammary carcinomas. It will be of interest to evaluate whether alterations of cytokines that are constitutively produced by mammary cells are also associated with high-grade tumours. © 1999 Cancer Research Campaign


*British Journal of Cancer* (1999) **80**(3/4), 579–584
© 1999 Cancer Research Campaign
Article no. bjoc.1998.0394

### Expression of interleukin 6 (IL-6) correlates with oestrogen receptor in human breast carcinoma

**G Fontanini[1], D Campani[1], M Roncella[2], D Cecchetti[1], S Calvo[1], A Toniolo[3] and F Basolo[1]**

Department of Oncology, Divisions of [1]Pathology and [2]Surgery, University of Pisa, 57 Via Roma, 56126, Pisa, Italy, [3]Department of Clinical and Biological Sciences, University of Pavia, Varese, Italy

**Summary** Multifunctional cytokines play important and only partially defined roles in mammary tumour development and progression. Normal human mammary epithelial cells constitutively produce interleukin 6 (IL-6), IL-8 and a non-secreted form of tumour necrosis factor. Transformation of mammary epithelial cells by different oncogenes is frequently associated with alterations of cytokine/growth factor production and responsiveness. In the present study we analysed the expression of IL-6 in 149 cases of invasive breast carcinoma and the data have been correlated with clinico-pathological variables including tumour size, histological grade, nodal status, and oestrogen and progesterone receptors, Ki67 and p53, protein expression. Though the majority of breast carcinomas expressed at least low levels of immunoreactive IL-6, we found that expression of this cytokine was inversely associated with histological tumour grade ($P = 0.0017$), but not with tumour size and nodal status. Ki67 positivity was inversely correlated with IL-6 expression ($P = 0.027$). Among biological parameters analysed, a direct association was found between the percentage of IL-6-positive cells and that of oestrogen ($P = 0.00005$) and progesterone ($P = 0.025$) receptor-positive cells. No correlation was observed between IL-6 and p53 protein expression. These data indicate that down-regulation of IL-6 is associated with highly malignant mammary carcinomas. It will be of interest to evaluate whether alterations of cytokines that are constitutively produced by mammary cells are also associated with high-grade tumours.

**Keywords:** cytokines; IL-6; breast cancer; ER; PgR; p53; Ki67

Interleukin 6 (IL-6) is a multifunctional regulator of immune responses, haemopoiesis, and acute phase reactions, as well as of cell growth and differentiation (Hirano, 1994). In addition, IL-6 de-regulation is also involved in a variety of diseases, including malignancies (plasmacytoma/myeloma, leukaemia/lymphoma, melanoma, Kaposi's sarcoma, renal cell carcinoma, salivary gland carcinoma), (Michiel et al, 1992). Production of IL-6 is up-regulated by cytokines such as IL-1 and tumour necrosis factor (TNF) (Hirano, 1994) as well as by certain oncogenes including *N-ras* (Castelli et al, 1994). In addition, it has been shown that the production of IL-6 is modulated by hormones, such as steroids (Ray and Prefortaine et al, 1994). These observations suggest that different pathways could lead to the altered expression of this cytokine.

Since cytokines seem to play an important role both in physiological and pathological conditions, we have previously analysed the cytokine pattern expression in cultures of human mammary epithelial cells (MEC) obtained from healthy donors as well as in cultures of immortalized and oncogene-transformed MEC. IL-6 expression has also been studied in sections obtained from normal mammary glands, and non-invasive and invasive carcinomas. Our results have shown that normal MEC produce IL-6 and IL-8 together with a non-secreted form of TNF (Basolo et al, 1993*a*). In addition, we found that MEC transformation by different oncogenes (*c-Ha-ras*, *c-erb-B2*, simian virus 40 T large antigen) is frequently associated with the loss of IL-6 production and responsiveness to exogenous IL-6 (Basolo et al, 1993*b*; Basolo et al, 1996*a*).

Immunohistochemical studies have also shown that, as compared to normal tissue and to in situ lesions, expression of immunoreactive IL-6 in human mammary tissue is significantly reduced in invasive ductal carcinoma (Basolo et al, 1996*b*). Parallel in vitro studies partially supported the in vivo findings: IL-6 expression was significantly reduced in cultures derived from both ductal carcinoma and peritumoral tissue. All these reports suggest that alterations of IL-6 pathways are a frequent event in human breast tumorigenesis.

Although there is some information on the possible role of IL-6 in plasmacytoma/myeloma, leukaemia/lymphoma, melanoma, Kaposi's sarcoma, renal cell and salivary gland carcinomas (Hirano, 1994; Gandour-Edwards et al, 1995*a*; Silvani et al, 1995) no data are available on the correlation between IL-6 expression and the clinico-pathological variables in mammary neoplasia. This has prompted us to study the expression of IL-6 in a series of breast carcinomas and to correlate the positivity for this cytokine to clinico-pathological and biological parameters.

### MATERIALS AND METHODS

### Patients

For this study, 149 cases were chosen among the patients diagnosed at the Department of Oncology of the S Chiara Hospital, University of Pisa. Selected patients had not received any antineoplastic treatments before surgery.

*Received 5 February 1998*
*Revised 20 May 1998*
*Accepted 27 May 1998*

*Correspondence to:* F Basolo

Supported by the Italian Association for Cancer Research (AIRC, Milan)

**Table 1** Association between IL-6 expression and clinico-pathological parameters in breast cancer

| Feature | IL-6 expression[a] | | | |
|---|---|---|---|---|
| | No | Negative | Positive | $P$[b] |
| Histotype | | | | |
| Ductal infiltrating | 121 | 20 | 101 | |
| Lobular infiltrating | 22 | 4 | 18 | |
| Medullary carcinoma | 6 | 2 | 4 | |
| Pathological tumour size | | | | |
| pT1 | 80 | 14 | 66 | |
| pT2–3 | 64 | 13 | 51 | NS |
| NK[c] | 5 | | | |
| Histological grading | | | | |
| I | 25 | 0 | 25 | |
| II/III | 96 | 20 | 76 | 0.0017 |
| No. of involved nodes | | | | |
| 0 | 79 | 17 | 62 | |
| < 3 | 32 | 4 | 28 | NS |
| > 3 | 31 | 5 | 26 | NS |
| NK | 7 | | | |

[a]Tumours with scores of 0 and 1 were considered as negative; tumours with scores ranging from 2 to 7 were considered as positive. [b]Statistical analysis: $\chi^2$ with Fisher's correction (the NK group as not included). [c]NK, not known.

**Table 2** Association between IL-6 expression and biological parameters in breast cancers

| Feature | IL-6 expression[a] | | | |
|---|---|---|---|---|
| | No | Negative | Positive | $P$[b] |
| p53 expression | | | | |
| Negative | 117 | 22 | 95 | |
| Positive | 33 | 5 | 28 | NS |
| ER expression[c] | | | | |
| Negative | 50 | 14 | 36 | |
| Positive | 94 | 11 | 83 | 0.02 |
| NK[d] | 5 | | | |
| PR expression[c] | | | | |
| Negative | 50 | 10 | 40 | |
| Positive | 52 | 5 | 47 | NS |
| NK | 47 | | | |
| Ki67 expression[e] | | | | |
| Low | 19 | 6 | 13 | |
| High | 44 | 6 | 38 | NS |
| NK | 86 | | | |

[a]Tumours with scores of 0 and 1 were considered as negative; tumours with scores ranging from 2 to 7 were considered as positive. [b]Statistical analysis: $\chi^2$ with Fisher's correction (the NK group was not included). [c]Tumours with ≤5% positive cells were considered as negative; tumours with > 5% positive cells were considered as positive. [d]NK, not known. [e]Low expression means ≤10% of positive cells; high expression, means > 10% of cells.

### IL-6 and p53 protein expression

After fixation in 10% formalin, processing and paraffin embedding, 3- to 5 μm-thick sections were prepared. Sections were dewaxed and dehydrated, and the endogenous peroxidase activity was blocked by incubation for 15 min with 0.3% hydrogen peroxide in methanol. Sections were stained with two different anti-IL-6 antibodies (rabbit polyclonal antibody and IL-6 mouse IgG1 monoclonal antibody, Genzyme Co., Cambridge, MA, USA; product codes LP-716 and 1618-01), p53 mouse IgG2b monoclonal antibody (DAKO A/S, Denmark; clone DO7). Antibody dilutions (1:100 or 1:500) were incubated overnight; immunoreactivity was revealed with the avidin–biotin immunoperoxidase complex technique (Vector Elite ABC kit; Vector Laboratories, Burlingame, CA, USA). Sections were scored independently by two pathologists (GF and FB) using the following method: (a) the intensity of staining for each antigen was scored from 0 to 3 (0, absent; 1, weak; 2, moderate; and 3, strong); (b) the proportion of malignant cells positively stained was scored from 0 to 4 (0, no positive cells; 1, < 10% positive cells; 2, 11–50%; 3, 51–75%; and 4, 76–100%). The two scores were then added to yield the total score (0–7). Immunoreactivity was defined as negative when the score was 0, weak when it was 1–3, and strong when it was > 4. Tumours with scores ranging from 2 to 7 were considered as positive. Appropriate controls were included in every experiment. Previous studies (Basolo et al, 1993*a*) had shown that the sensitivity of immunostaining for IL-6 with the two antibodies used in this study was high. We have now shown that cytoplasmic positivity is maintained even in cultured cells releasing as little as 100–200 pg of IL-6 per $10^6$ cells. As specificity controls, we showed that immunostaining of mammary tissue sections by IL-6 antibodies was blocked by incubation with recombinant IL-6 (Genzyme; 300 ng cytokine + 1 μg antibody in 100 μl for 6 h), but not with equivalent amounts of recombinant IL-2 or IL-8 (Genzyme).

### Ki67 labelling index

The nuclear proliferation-associated antigen Ki67 was assayed on frozen sections using the Ki67 mouse monoclonal antibody (Dako A/S) diluted 1:10 in phosphate-buffered saline (PBS) and incubated overnight. Immunoreactivity was revealed as stated above. Negative control sections were incubated with normal mouse serum. As previously reported (Campani et al, 1991), the evaluation of Ki67 was expressed as the percentage of positive cells. For each tumour section, at least 25 microscopic fields (40 × objective) were analysed. The percentage was calculated dividing the number of Ki67-positive neoplastic cells by the total number of cells counted, and the result multiplied by 100. Tumours were classified as highly proliferating if more than 10% (median value of the distribution) of cells were immunoreactive (Campani et al, 1991).

### Hormone receptor analysis

The presence of oestrogen receptor (ER) was determined using the Abbott ER-ICA monoclonal kit (Abbott Laboratories, Abbott Park, MI, USA), as described (Campani et al, 1991). Briefly, rat anti-human ER antibody was incubated with frozen sections followed by a goat anti-rat IgG and revealed with a rat peroxidase–anti-peroxidase complex. Peroxidase activity was detected by the incubation of the antibody complex with 3,3′-diaminobenzidine and hydrogen peroxide. Expression of progesterone receptor (PR) was determined by the same method as ER, using the Abbott PR-ICA monoclonal kit (Abbott Laboratories). Negative controls were incubated with normal rat serum. ER and PR expression was quantitated as a percentage value as described for Ki67. Tumours with ≤ 5% positive cells were classified as negative.

© Cancer Research Campaign 1999

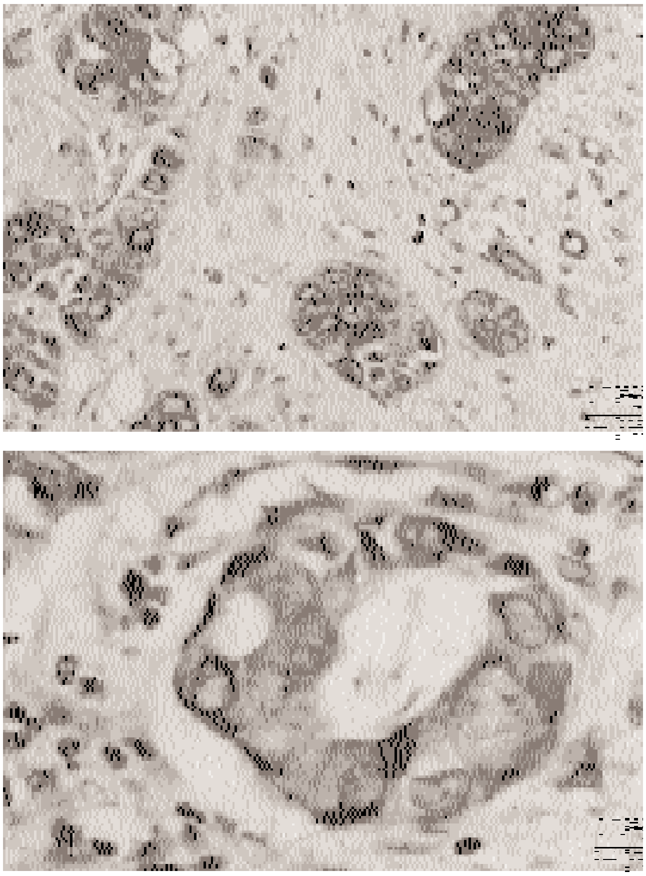

**Figure 1** Immunohistochemical staining for IL-6 in invasive ductal carcinoma. (**A**) Strong cytoplasmic immunoreactivity for IL-6 in the neoplastic component of epithelial cells (original magnification, ×250). (**B**) High magnification of neoplastic gland showing strong and specific immunoreactivity for IL-6 (original magnification, ×400)

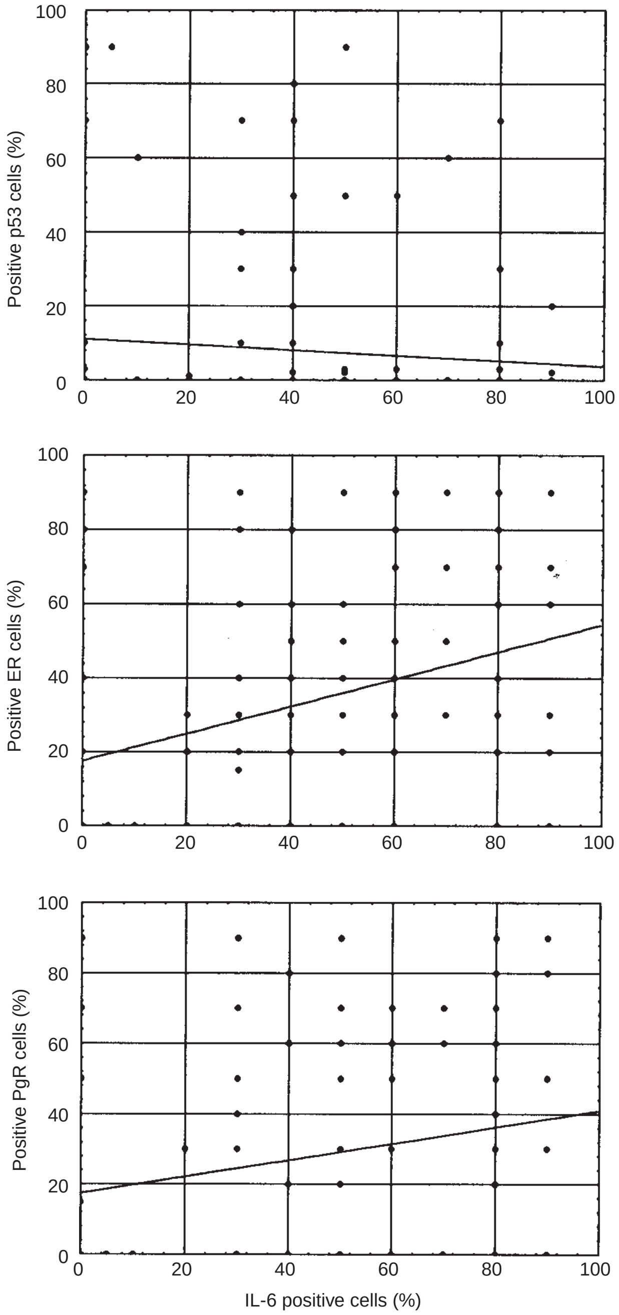

**Figure 2** Correlation between IL-6 expression and expression of p53, PR and ER in breast carcinoma. The percentage of IL-6-positive cells in each case was related to the positivity for p53, PR or ER. Statistical analysis was performed by the Spearman test for continuous variables. No correlation was found between IL-6 and p53 expression ($r = -0.042$, $t = -0.51$; $P = 0.6$); a direct correlation was observed between IL-6 expression and positivity for both ER ($r = 0.381$, $t = 3.7$, $P = 0.00037$) and PR ($r = 0.22$, $t = 2.27$; $P = 0.025$)

### Histological grading

Cases selected for this study were reviewed by two pathologists (FB and GF) and classified according to a recent modification of the WHO Histological Classification of Breast Tumours (Rosen and Oberman, 1993). The Scarff, Bloom and Richardson histo-prognostic grade was scored according to the Contesso recommendations (Contesso et al, 1987) by evaluating three parameters: tubular differentiation (throughout, 1; occasional, 2; not seen, 3), nuclear polymorphism (uniform and regular size, 1; moderate pleomorphism, 2; very pleomorphic, 3) and mitotic index (1 mitosis, 1; 2, 2; 3, 3). The final grade is determined by adding the three scores: grade 1, 3–5; grade 2, 6–7; grade 3, 8–9.

### Statistical methods

A $\chi^2$ test with Fisher's correction was used for the correlating clinico-pathological parameters with IL-6 immunoreactivity. For continuous variables, Sperman's test was used to analyse the correlation between the percentages of IL-6-positive cells and positivity for p53, ER, PR and K67.

### RESULTS

### IL-6 protein expression

The histotype of all cases was determined according to the widely accepted criteria of Rosen and Oberman (Rosen and Oberman, 1993). IL-6 expression was studied by immunostaining with polyclonal and monoclonal antibodies. Both reagents produced clear

© Cancer Research Campaign 1999

cytoplasmic reactivity, but the staining pattern produced by the polyclonal rabbit antibody was more intense and reproducible (Figure 1 A, B). Reactivity of both antibodies was totally abolished by preincubation with recombinant-IL-6 (r-IL-6), but not with either r-IL-2 or r-IL-8. The immunoreactivity of each tumour was scored from 0 to 7 as reported in Materials and Methods. The staining pattern was heterogeneous (i.e. in positive sections the intensity of staining ranged from negative to strong). As reported in Table 1, the majority of cases expressed immunoreactive IL-6 (82%). Among cases of IL-6-negative carcinoma, staining was clearly limited to remaining normal ducts and lobules.

### IL-6 expression in relation to clinico-pathological parameters

In Table 1, patients were grouped according to IL-6-negativity and IL-6-positivity. We analysed the data in relation to the following clinico-pathological variables: tumour size, histological grade and nodal status. A significant association was found between IL-6 expression and histological grading of the tumours; i.e. low-grade tumours (grade I) had higher IL-6 expression than high-grade tumours (grade II and III; $P = 0.0017$). No statistically significant association was, however, found between IL-6 expression and tumour size or nodal status.

### IL-6 expression in relation to p53, ER, PR and Ki67

p53 overexpression was observed in 33 (22%) out of 149 carcinomas analysed. However, no differences were observed in IL-6 expression between p53-positive and p53-negative cases (Table 2). No correlation was detected by the Sperman's test between p53 and IL-6 expression ($r = -0.042$, $t = -0.51$, $P = 0.6$). Interestingly, among the 94 ER-positive carcinomas, 83 (88%) cases were IL-6-positive, while among the 50 ER-negative tumours 36 (72%) were IL-6-positive ($P = 0.02$). This conclusion was confirmed by the Sperman analysis that showed a significant direct correlation between the percentage of IL-6-positive cells and the percentage of ER-positive cells ($r = 0.381$, $t = 3.7$, $P = 0.00037$). A significant direct correlation was also found between PR and IL-6 expression ($r = 0.22$, $t = 2.27$; $P = 0.025$). On the contrary, an inverse correlation was observed between IL-6 expression and the proliferative index, as evaluated by Ki67 immunoreactivity ($r = -0.28$, $t = -2.28$, $P = 0.027$). Figure 2 summarizes the statistical correlation between IL-6, ER, PR and p53 expression.

### DISCUSSION

Over the last few years, the role of cytokines in cancer has been the subject of numerous investigations (Porter and Lippman, 1994). It has been reported that, in the mammary gland, cytokines play a role in growth and differentiation (Massagué et al, 1992; Basolo et al, 1993*b*; Danforth and Sagias, 1993), extracellular matrix production (Silberstein et al, 1992), angiogenesis (Garver et al, 1994) and as immunomodulating factors (Allione et al, 1994). Recently, we have shown that epithelial cells of the normal mammary gland produce constitutively IL-6, IL-8 and a non-secreted form of TNF (Basolo et al, 1993*a*). In addition, we observed that upon transformation in vitro by several different oncogenes (except for *int-2*), MEC lose the ability of producing and responding to IL-6 (Basolo et al, 1993*b*).

It has also been demonstrated in vivo that, as compared to normal mammary tissue, the expression of IL-6 in neoplastic tissues is slightly reduced in non-invasive ductal carcinoma, but strongly reduced in the invasive forms of ductal and medullary carcinoma. This finding suggests an inverse relationship between tumour aggressiveness and IL-6 expression, in agreement with observations in tumours of the salivary gland (Gandour-Edwards et al, 1995*a*) and thyroid (Basolo et al, 1998). These results have been confirmed by experiments in vitro. Primary cultures from ductal carcinomas produce reduced amounts of IL-6 as compared to primary cultures obtained from the normal mammary gland (Basolo et al, 1996*b*).

IL-6 expression appears to relate to prognosis in tumours other than those of breast. An inverse relationship between IL-6 expression and biological aggressiveness has been reported in salivary gland tumours (Gandour-Edwards et al, 1995*a*), malignant melanoma cells have been shown to be unresponsive to exogenous IL-6 (Lu and Kerbel, 1993; Silvani et al, 1995), invasive pituitary malignancies appear to overexpress IL-6 (Gandour-Edwards et al, 1995*b*). In ovarian cancer patients, IL-6 serum levels have been correlated with a poor prognosis, since patients with low IL-6 levels had better survival than patients with high levels of this cytokine (Scambia et al, 1995). The above results prompted us to investigate whether alterations of IL-6 expression were correlated with tumour aggressiveness as evaluated by clinico-pathological and biological parameters. Although no association was observed between IL-6 expression and tumour size or nodal status, a statistically significant association was found with the histological grade of tumours. Approximately one-fifth (25/121) of investigated ductal carcinomas were classified as grade I. None of these 25 well-differentiated tumours was IL-6-negative. On the contrary, 20/96 (21%) grade II or III ductal carcinomas were IL-6-negative. Since high grade indicates less differentiated tumours, this finding suggests that reductions of IL-6 expression are associated with late stages of tumorigenesis. This was confirmed by the observation that highly proliferating tumours (as evaluated by Ki67 immunoreactivity) were characterized by low percentages of IL-6-positive cells ($r = -0.28$, $t = -2.28$, $P = 0.027$). Other interesting correlations of IL-6 expression with other biological parameters (ER, PR) were detected.

On the contrary, IL-6 expression was directly correlated with the expression of ERs and PRs that represent important mammary differentiation markers. Eighty-three out of 94 (88%) ER-positive tumours were also reactive with antibody to IL-6 ($P = 0.02$; Table 2), and the percentage of ER-positive cells was directly related to that of IL-6-positive cells ($P = 0.00005$; Figure 2). Equivalent results were obtained with regard to the percentages of PR-positive cells ($P = 0.025$; Figure 2). The potential interaction between cytokines and steroid hormones has already been suggested by studies of breast cyst fluid (Reed et al, 1992). It was observed that IL-1 and IL-6 do increase oestrogen synthesis by stimulating aromatase and oestradiol-dehydrogenase activities in breast cancer cells. In addition, it has been reported that ER-positive human breast cancer cells express the IL-6 receptor and that their proliferation is inhibited by addition of IL-6 (Chen et al, 1988; Chiu et al, 1996). In experiments in vitro we found that insertion of the *int*-2 gene into MEC results in the up-regulation of IL-6-receptor thus promoting cell proliferation in response to exogenous IL-6 and glucocorticoids (Basolo et al, 1993*a*). Taken together, these results indicate a possible autocrine and/or paracrine role of IL-6 at least

 © Cancer Research Campaign 1999

in the subgroup of mammary tumours that express the IL-6-receptor. The existence of a complex interplay between IL-6 pathways and steroid hormones is further exemplified by the finding that in human osteoblasts the IL-6 promoter is inhibited by oestrogen in the absence of a functional ER binding site (Stein and Yang, 1995). In conclusion, it appears that IL-6 is capable of modulating steroid hormone responsiveness and vice-versa.

Overexpression of the p53 protein has been correlated with poor patient prognosis in breast cancer (Stenmark-Askmalm et al, 1995; Horne et al, 1996), indicating that p53 represents an important prognostic marker. However, in this study we failed to detect correlations between p53 overexpression and IL-6 expression (Table 2, $P = 0.2$; Figure 2, $P = 0.6$). It is of interest to note that p53 appears to modulate IL-6 expression. Experiments in HeLa cells demonstrated that the IL-6 promoter is repressed by wild-type p53 and up-regulated by mutant forms (Margulies et al, 1993; Stenmark-Askmalm et al, 1995).

Since MEC constitutively produce not only IL-6, but also IL-8 and a non-secreted form of TNF, further studies are needed to ascertain if the expression of the ER and PR differentiation markers is also associated with altered expression of the above cytokines. In this context it should be recalled that Fas (APO-1/CD95) is expressed on the surface of normal and transformed MEC (Keane et al, 1996) and that interaction of Fas with ligands of the TNF family may trigger programmed cell death (Hug, 1997). It will be of interest to evaluate whether Fas expression is downregulated in high grade mammary tumours as a means of escaping the apoptotic response as already reported for colon carcinoma (von Reyhet et al, 1998).

### ACKNOWLEDGEMENTS

We thank ME Sobel, Department of Pathology, National Cancer Institute, NIH, Bethesda, MD, USA, for his critical review of the manuscript.

### REFERENCES

Allione A, Consalvo M, Nanni P, Lollini PL, Cavallo F, Giovarelli M, Forni M, Gulino A, Colombo MP, Dellabona P, Hock H, Blankestein T, Rosenthal FM, Gansbacher B, Bosco MC, Musso T, Gusella L and Forni G (1994) Immunizing and curative potential of replicating and nonreplicating murine mammary adenocarcinoma cells engineered with interleukin (IL) -2, IL-4, IL-6, IL-7, IL-10, tumor necrosis factor alpha, granulocyte-macrophage colony-stimulating factor, and gamma-interferon gene or admixed with conventional adjuvants. *Cancer Res* **54**: 6022–6026

Basolo F, Conaldi PG, Fiore L, Calvo S and Toniolo A (1993*a*) Normal breast epithelial cells produce interleukin 6 and 8 together with tumor-necrosis factor: defective IL-6 expression in mammary carcinoma. *Int J Cancer* **55**: 1–5

Basolo F, Calvo S, Fiore L, Conaldi PG, Falcone V and Toniolo A (1993*b*) Growth-stimulating activity of interleukin 6 (IL-6) on human mammary epithelial cells transfected with the *int-2* gene. *Cancer Res* **53**: 2957–2960

Basolo F, Fiore L, Calvo S, Falcone V, Conaldi PG, Fontanini G, Caligo AM, Merlo G, Gluzman Y and Toniolo A (1996*a*) Defective IL-6 expression and responsiveness in human mammary cells transformed by an adeno-5/sv40 hybrid virus. *Br J. Cancer* **73**: 1356–1361

Basolo F, Fiore L, Fontanini G, Conaldi PG, Calvo S, Falcone V and Toniolo A (1996*b*) Expression of interleukin 6 (IL-6) and response to it in human mammary tumours. *Cancer Res* **56**: 3118–3122

Basolo F, Fiore L, Pollina L, Fontanini G, Conaldi PG and Toniolo A (1998) Reduced expression of interleukin 6 in undifferentiated thyroid carcinoma: in vitro and in vivo studies. *Clin Cancer Res* **4**: 381–387

Campani D, De Negri F, Martini L, Giani C, Squartini F and Sarnelli R (1991) Estrogen, progesterone receptors and proliferating activity evaluated by immunocytochemistry in breast cancer. *Int J Biol Markers*, **6**: 144–150

Castelli C, Sensi M, Lupetti R, Mortarini R, Panceri P, Anichini A and Parmiani G (1994) Expression of interleukin 1α, interleukin 6, and tumour necrosis factor α genes in human melanoma clones is associated with that of mutated *N-ras* oncogene. *Cancer Res* **54**: 4785–4790

Chen I, Mory Y, Zilberstein A and Revel M (1988) Growth inhibition of human breast carcinoma and leukemia/lymphoma cell lines by recombinant interferon-β. *Proc Natl Acad Sci USA*, **85**: 8037–8041

Chiu JJ, Sgagias MK and Cowan KH (1996) Interleukin 6 acts as a paracrine growth factor in human mammary carcinoma cell lines. *Clin Cancer Res* **2**: 215–221

Contesso G, Mouriesse H, Friedman S, Genin J, Sarrazin D and Rouesse J (1987) The importance of histologic grade in long-term prognosis of breast cancer: a study of 1010 patients, uniformly treated at the Institut Gustave Roussy. *J Clin Oncol* **5**: 1378–1386

Danforth DN and Sagias MK (1993) Interleukin-1 alpha and interleukin-6 act additively to inhibit growth of MCF-7 breast cancer cells in vitro. *Cancer Res* **53**: 1538–1545

Gandour-Edwards R, Kapadia SB, Gumerlock PH and Barnes AL (1995*a*) Immunolocalization of interleukin-6 in salivary gland tumors. *Hum Pathol* **26**: 501–503

Gandour-Edwards R, Kapadia SB, Janecka IP, Martinez AJ and Barnes L (1995*b*) Biological markers of invasive pituitary adenomas involving the sphenoid sinus. *Mod Pathol* **8**: 160–164

Garver RI, Radford DM, Donis-Keller H, Wick MR and Milner PG (1994) Midkine and pleiotrophin expression in normal and malignant breast tissue. *Cancer* **74**: 1584–1590

Hirano T (1994) Interleukin-6. In *The Cytokine Handbook,* Thomson A (ed), pp. 145–168. Academic Press: London

Horne GM, Anderson JJ, Tiniakos DG, McIntosh GG, Thomas MD, Angus B, Henry JA, Lennard TWJ and Horne CHW (1996) p53 protein as a prognostic indicator in breast carcinoma: a comparison of four antibodies for immunohistochemistry. *Br J Cancer* **73**: 29–36

Hug H (1997) Fas-mediated apoptosis in tumor formation and defense. *Biol Chem* **378**: 1405–1412

Keane MM, Ettemberg SA, Lowrey GA, Russel EK and Lipkowitz S (1996) Fas expression and function in normal and malignant breast cell lines. *Cancer Res* **56**: 4791–4798

Lu C and Kerbel RS (1993) Interleukin-6 undergoes transition from paracrine growth inhibitor to autocrine stimulator during human melanoma progression. *J Cell Biol* **120**: 1281–1288

Margulies L and Sehgal B (1993) Modulation of the Interleukin-6 promoter (IL-6) and transcription factor C/EBPb /NF-IL-6) activity by p53 species. *J Biol Chem* **268**: 15096–15100

Massagué J, Cheifetz S, Laiho M, Ralph DA, Weis FMB and Zentella A (1992) Transforming growth factor-β. In *Tumor Suppressor Genes, the Cell Cycle and Cancer,* Levine AJ (ed), pp. 81–103. Cold Spring Harbor Laboratory Press: Cold Spring Harbor, NY

Michiel DF and Oppenheim JJ (1992) Cytokines as positive and negative regulators of tumor promotion and progression. *Semin Cancer Biol* **3**: 3–15

Porter JK and Lippman ME (1994) Overview of the biologic markers of breast cancer. *Hematol Oncol Clin North Am*, **8**: 73–100

Ray A and Prefontaine KE (1994) Physical association and functional antagonism between the p65 subunit of transcription factor NF-kappa B and the glucocorticoid receptor. *Proc Natl Acad Sci USA* **91**: 752–756

Reed MJ, Coldham NG, Patel SR, Ghilcchick MW and James VHT (1992) Interleukin-1 and interleukin-6 in breast cyst fluid: their role in regulating aromatase activity in breast cancer cells. *J Endocrinol* **132**: R5–R8

Rosen PP and Oberman HA (1993) Tumors of the mammary gland. In *Atlas of Tumor Pathology,* Rosai J (ed), pp. 7–10. Armed Forces Institute of Pathology: Washington, DC

Scambia G, Testa U, Benedetti Panici P, Foti E, Martucci R, Gadducci A, Perillo A, Facchini V, Peschle C and Mancuso S (1995) Prognostic significance of interleukin 6 serum levels in patients with ovarian cancer. *Br J Cancer* **71**: 354–356

Silberstain GB, Flanders KC, Roberts AB and Daniel CW (1992) Regulation of mammary morphogenesis: evidence for extracellular matrix-mediated inhibition of ductal budding by transforming growth factor-β 1. *Dev Biol* **152**: 354–362

Silvani A, Ferrari G, Paonessa G, Toniatti C, Parmiani G and Colombo MP (1995) Down-regulation of interleukin 6 receptor α chain in interleukin 6 transduced melanoma cells causes selective resistance to interleukin 6 but not to oncostatin M. *Cancer Res* **55**: 2200–2205

Stein B and Yang M (1995) Repression of the interleukin-6 promoter by estrogen receptor is mediated by NF-κB and C/EBPb. *Mol and Cellular Biol* 4971–4979

© Cancer Research Campaign 1999 

Stenmark-Askmalm M, Stal O, Olsen K and Nordenskjold B (1995) p53 as a prognostic factor in stage I breast cancer. South-East Sweden Breast Cancer Group. *Br J Cancer* **72**: 715–719

von Reyher U, Strater J, Kittstein W, Gschwendt M, Krammer PH and Moller P (1998) Colon carcinoma cells use different mechanisms to escape CD95-mediated apoptosis. *Cancer Res* **58**: 526–534

*© Cancer Research Campaign 1999*
